# Supplementary material for: The Expression of Active CD11b Monocytes in Blood and Disease Progression in Amyotrophic Lateral Sclerosis
Source: Int J Mol Sci. 2022 Mar 21;23(6):3370. doi: 10.3390/ijms23063370 (PMC8952310; doi:10.3390/ijms23063370)

Supplementary Figure S1.

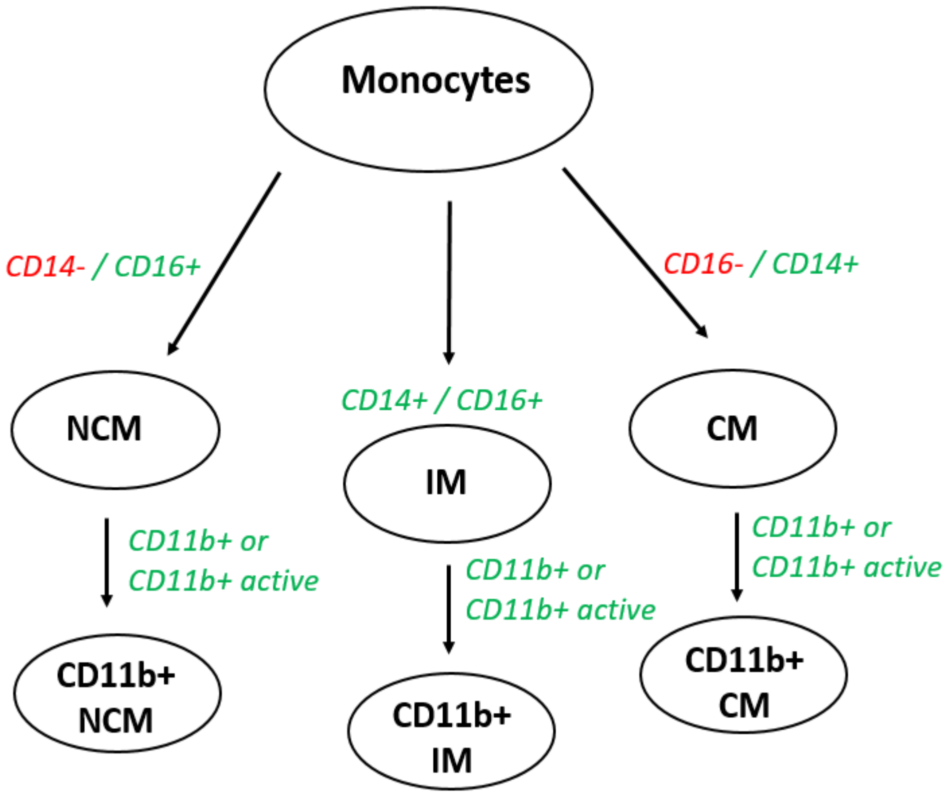

Supplementary Figure S2.

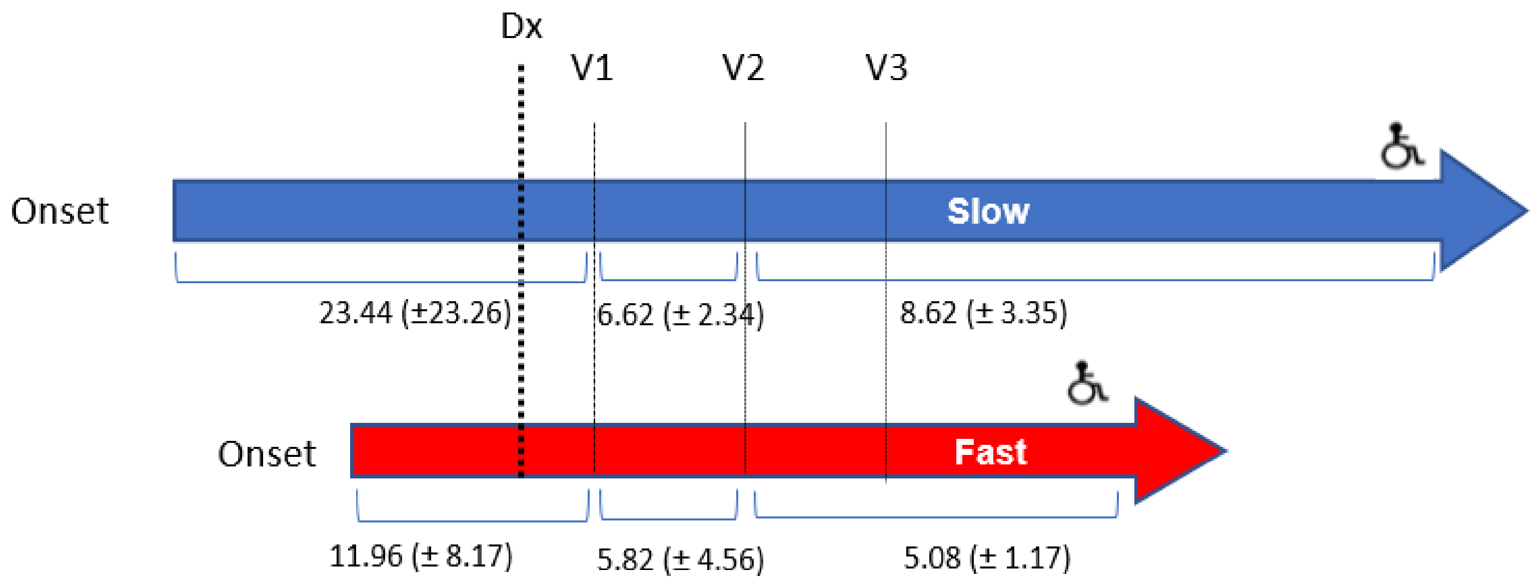

Supplementary Figure S3.

A.

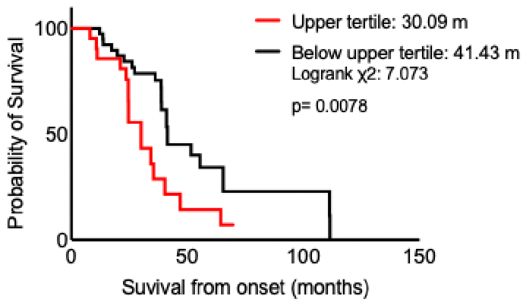

B.

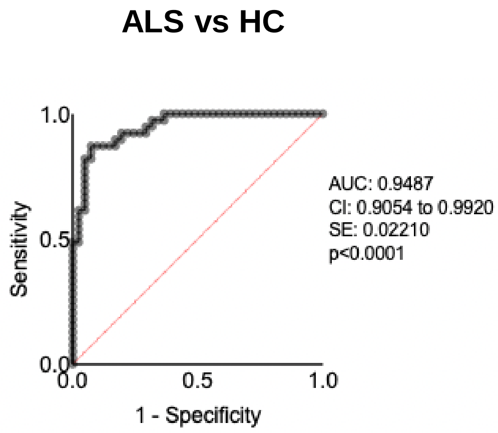

Supplementary Figure S4.

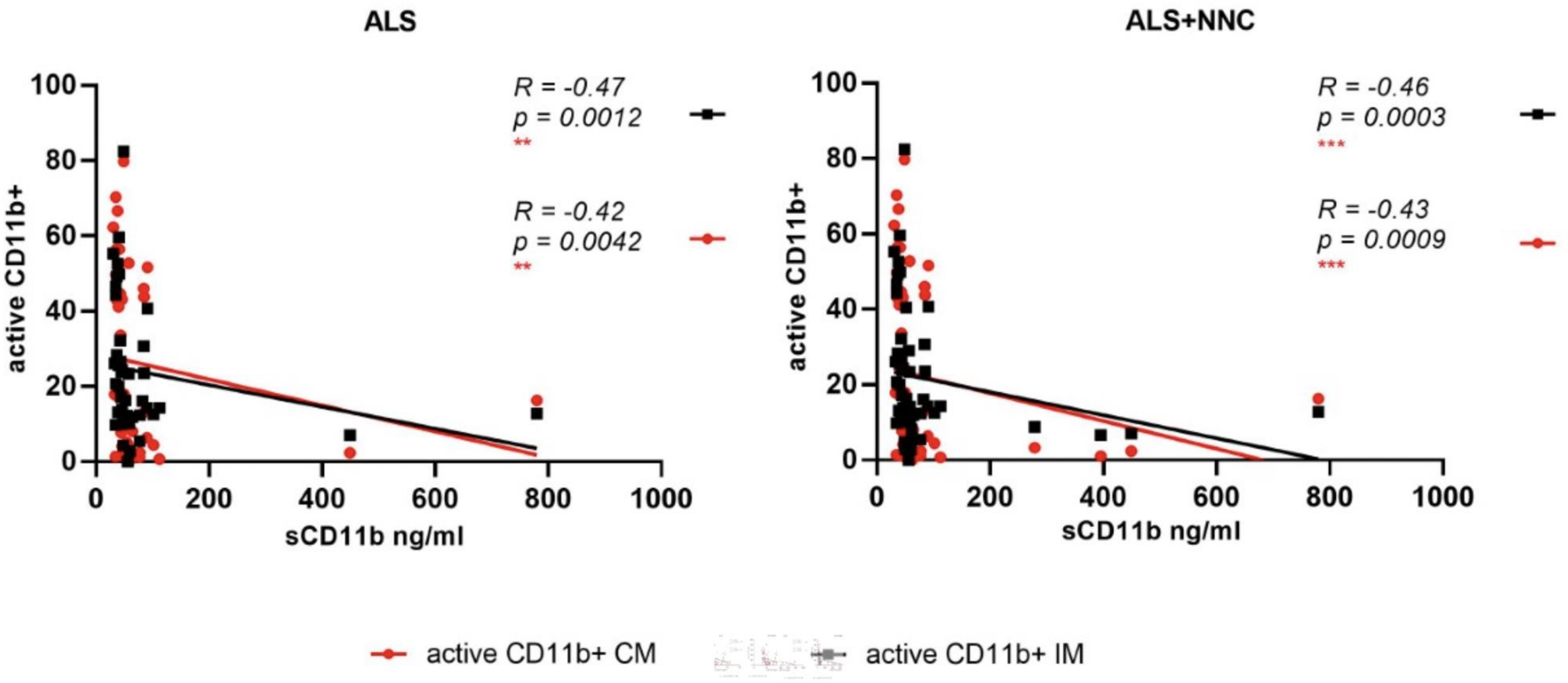

Supplement: Supplementary file 1 [file ijms-23-03370-s001.zip › Figure S1-S4.pdf]
