# Supplementary material for: The Expression of Active CD11b Monocytes in Blood and Disease Progression in Amyotrophic Lateral Sclerosis
Source: Int J Mol Sci. 2022 Mar 21;23(6):3370. doi: 10.3390/ijms23063370 (PMC8952310; doi:10.3390/ijms23063370)
Supplement: Supplementary file 1 [file ijms-23-03370-s001.zip › Table S1.pdf]

Baseline	Age	ALSFRS-R	Survival V1	Survival onset	ΔFRS	ALSFRS-R change
HLA-DR+	*P= 0.0170 R= 0.3850	P= 0.9598 R= 0.0084	**P= 0.0038 R= -0.4588	P= 0.5733 R= -0.0943	P= 0.1883 R= 0.2181	P= 0.8738 R= -0.0266
CD11b+NCM	*P=0.0100 R=0.4128	P= 0.6016 R= -0.0874	**P= 0.0086 R= -0.4203	P= 0.9140 R= 0.01812	P= 0.0065 R= 0.4342	*P= 0.0180 R= 0.3819
CD11b+IM	***P=0.0003 R=0.5593	P= 0.5060 R= 0.1113	*P= 0.0260 R= -0.3609	P= 0.9289 R= 0.0149	P= 0.8500 R= -0.03173	P= 0.7618 R= 0.0508
Active CD11b+NCM	**P= 0.0015 R= -0.4964	P= 0.8664 R= 0.0282	*P= 0.0166 R= 0.3862	P= 0.9721 R= -0.0058	P= 0.5722 R= -0.0945	P= 0.8396 R= -0.0339
Active CD11b+IM	P= 0.7513 R= 0.0531	P= 0.8580 R= 0.0300	P= 0.7333 R= -0.0571	P= 0.5316 R= -0.1047	P= 0.8802 R= 0.0253	P= 0.1607 R= 0.2322
Active CD11b+CM	P= 0.7581 R= -0.0516	P= 0.4518 R= -0.1258	P= 0.8300 R= 0.0360	P= 0.5698 R= -0.0951	P= 0.3945 R= 0.1422	P= 0.0571 R= 0.3114

Table S1. Pairwise correlation analysis of associations between monocyte subsets expression at baseline and age at V1 and clinical variables of disease progression. Significant associations are highlighted in grey. Log-transformed monocyte frequencies are used for analysis.
